# Precision Mapping of Intrahepatic Biliary Anatomy and Its Anatomical Variants Having a Normal Liver Using 2D and 3D MRCP

**DOI:** 10.3390/diagnostics13040726

**Published:** 2023-02-14

**Authors:** Jehan A. Mazroua, Yassir Edrees Almalki, Mohamed Alaa, Sharifa Khalid Alduraibi, Mervat Aboualkheir, Asim S. Aldhilan, Ziyad A. Almushayti, Sameh Abdelaziz Aly, Mohammad Abd Alkhalik Basha

**Affiliations:** 1 Department of Diagnostic Radiology, Faculty of Human Medicine, Mansoura University, Mansoura 35516, Egypt; 2 Division of Radiology, Department of Internal Medicine, Medical College, Najran University, Najran 61441, Saudi Arabia; 3 Department of Radiology, College of Medicine, Qassim University, Buraidah 52571, Saudi Arabia; 4 Department of Radiology and Medical Imaging, College of Medicine, Taibah University, Madinah 42353, Saudi Arabia; 5 Department of Diagnostic Radiology, Faculty of Human Medicine, Benha University, Benha 13511, Egypt; 6 Department of Radiology, Faculty of Human Medicine, Zagazig University, Zagazig 44519, Egypt

**Keywords:** MRCP, intrahepatic biliary tree, intraoperative cholangiography

## Abstract

Despite significant advances in hepatobiliary surgery, biliary injury and leakage remain typical postoperative complications. Thus, a precise depiction of the intrahepatic biliary anatomy and anatomical variant is crucial in preoperative evaluation. This study aimed to evaluate the precision of 2D and 3D magnetic resonance cholangiopancreatography (MRCP) in exact mapping of intrahepatic biliary anatomy and its variants anatomically in subjects with normal liver using intraoperative cholangiography (IOC) as a reference standard. Thirty-five subjects with normal liver activity were imaged via IOC and 3D MRCP. The findings were compared and statistically analyzed. Type I was observed in 23 subjects using IOC and 22 using MRCP. Type II was evident in 4 subjects via IOC and 6 via MRCP. Type III was observed equally by both modalities (4 subjects). Both modalities observed type IV in 3 subjects. The unclassified type was observed in a single subject via IOC and was missed in 3D MRCP. Accurate detection by MRCP of intrahepatic biliary anatomy and its anatomical variants was made in 33 subjects out of 35, with an accuracy of 94.3% and a sensitivity of 100%. In the remaining two subjects, MRCP results provided a false-positive pattern of trifurcation. MRCP competently maps the standard biliary anatomy.

## 1. Introduction

Hepatobiliary surgery has become more common and complex; therefore, a precise assessment of the biliary anatomy is essential for the success and safety of these procedures [1,2,3]. Despite developments in hepatic surgery techniques, complications of biliary anatomy remain the chief cause of mortality and morbidity [4,5,6,7]. The aim is to select the optimal treatment method, reduce the likelihood of surgical problems, and pinpoint anatomical regions that require careful attention during the operation [8]. Comprehensive knowledge of the anatomic variations of the hepatic biliary system is essential for surgical planning and for reducing the risk of postoperative complications [1,2,3,4,5,6,7,8]. 

Several imaging modalities can be used to determine the biliary anatomy prior to surgery, including magnetic resonance cholangiopancreatography (MRCP), computed tomography (CT) cholangiography, endoscopic retrograde cholangiography (ERCP), and percutaneous cholangiography (PC) [7,8,9,10,11,12]. As a non-invasive technique, MRCP has replaced intraoperative cholangiography (IOC) and ERCP for the evaluation of hepatic biliary anatomy. MRCP is useful for determining the optimal hepatectomy plane and identifying patients who require additional surgical intervention [7,8,13]. Three-dimensional (3D) MRCP is a new pulse sequence that produces ideal images for multiplanar reformations and provides an anatomical overview similar to that of conventional thick-slab MR images [14]. 3D MRCP has many advantages over traditional 2D imaging, including operator-independent imaging, thinner sections without interslice gaps, greater coverage volume, and a higher signal-to-noise ratio (SNR). However, blurring and ghosting artifacts are quite prominent in 3D MRCP imaging compared to 2D imaging due to the longer acquisition time and variability in the respiration depth of the patient associated with 3D MRCP imaging [15]. 3D MRCP appears to be an exceptional predictor of actual anatomy, achieving 100% sensitivity for typical biliary anatomy (type I) [16].

The present study evaluated the accuracy of 3D MRCP in mapping intrahepatic biliary anatomy and its anatomical variants compared to IOC in subjects with normal and healthy livers (living liver donors). 

## 2. Materials and Methods 

### 2.1. Statement of Ethics 

Approval for ethics was obtained from the local research ethics committee (approval no. 442-42-52706-DS; approved 2 May 2021). Written consent was obtained from all potential donors. The present research followed the Declaration of Helsinki’s ethical principles. 

### 2.2. Study Population 

Initially, 49 consecutive subjects were included in the study. The inclusion criteria were subjects with a normal liver chosen from liver donors after the exclusion of liver pathology and the completion of the pre-transplantation workup by the radiological transplantation team. Exclusion criteria were focal or diffuse hepatic lesions, such as hepatocellular carcinoma (*n* = 4), hepatic nodules (*n* = 5), periportal fibrosis (*n* = 2), and severe and diffuse hepatic steatosis (*n* = 3). The exclusion process resulted in a final cohort composed of 35 subjects. 

### 2.3. Subject Assessment 

At the gastroenterology center, the donors were carefully evaluated through clinical examination, laboratory tests, preoperative evaluation, and their relationship to the recipient. All donors underwent abdominal ultrasound (grayscale and color Doppler), liver biopsy (US-guided), CT angiography of the portal vein, hepatic veins, hepatic artery, plain X-ray of the chest, and CT volumetry to assess the graft size of the right lobe and residual volume of the donor liver. The healthy subjects proceeded to the MR unit to perform MRCP within one week. All potential donors underwent a conventional MRCP without using a contrast agent. 

### 2.4. Preparation of MRI 

Fasting for the last 6 h before the study was required to reduce fluid secretions within the duodenum and the stomach, promote gallbladder distension, and reduce bowel peristalsis. No contrast agent (oral or intravenous) was administered during the examination. 

### 2.5. Technique of MRCP 

The subjects were positioned on a movable examination table. Straps were used to help the subject stay still and maintain the right position throughout the imaging. MRCP inspections were performed on a 1.5-T closed MRI unit (Philips Ingenia closed wide bore) with an eight-channel phased-array body coil, circularly polarized, using the following: (i) A multiplanar fast-field echo (FFE) localizer, on which the pulse sequences were planned, beginning with the diaphragm and moving on to the lower border of the kidneys, with a slice thickness of 9 mm. (ii) A 2D slim superior labrum anterior to posterior (SLAP) and axial T2 single-shot fast spin-echo (SSFSE) respiratory-triggered sequence. The scan was initiated from the liver and moved down to the duodenum’s second part (the ampulla of Vater). (iii) A 3D slim SLAP coronal oblique with a heavy T2 fat-saturated fast-spin-echo (FAT SAT FSE) respiratory-triggered sequence. SLAPs were arranged parallel to the CBD to visualize both hepatic ducts. (iv) A 2D thin SLAP coronal T2 single-shot fast spin-echo (SSFSE) respiratory-triggered sequence. (v) A 2D coronal thick SLAP MYELO breath-hold sequence with 15 to 20 SLAPs centered on the CBD. The respiratory-triggered technique was used in the three primary pulse sequences to control motion artifacts. The total scan time ranged from 5 min and 50 s to 6 min and 10 s. The parameters of the MRCP protocol are described in Table 1. 

### 2.6. Processing of Image 

The imaging data obtained from the scans were studied on a workstation with 3D and 2D competence and multiple editing options. The reconstruction of the image and postprocessing of the source images of MRCP were achieved using an image of maximum intensity projection (MIP) formed in the coronal plane that showed the entire anatomy of the biliary system. Three-dimensional models of the hepatic ducts and common bile ducts were produced via a technique known as volume rendering (VR). The MIP and VR images were projected and magnified at a suitable angle to view the minor capacity of the standard common bile duct and intrahepatic bile ducts. A distinct consideration was given to the intrahepatic biliary radicles, especially the insertion of the right posterior sectoral duct, since it is considered the most crucial abnormality for optimal visualization. After the MIP and 3D VR images were obtained, the source images of the thin coronal sections and native axial sections were reviewed, which allowed for the optimum assessment of small accessory bile ducts or any of the small accessory bile duct branches. 

### 2.7. Interpretation and Analysis of Image 

The MRCP image analysis focused on the following criteria: (i) Identification of the joint intrahepatic biliary anatomy. (ii) Identification of intrahepatic biliary variants. (iii) Absence or presence of accessory bile ducts. We followed Choi et al.’s description [14] regarding intrahepatic biliary anatomy:

− Type I: The joint intrahepatic biliary anatomy consists of a horizontally oriented right posterior (RP) duct (draining segments VI and VII) joining the vertically oriented right anterior (RA) duct (draining segments V and VIII), forming the right hepatic duct (RHD), which ultimately connects to the left hepatic duct (LHD) (draining segments II, III, and IV), forming the common hepatic duct (CHD).
− Type II: trifurcation.
− Type III: anomalous RPSD insertion.
  - opens into the LHD.
  - opens into CHD (proximal to the cystic duct).
  - joins the cystic duct.
− Type IV: RHD inserts into the cystic duct.
− Type V: type I + Presence of an accessory duct.
  - type I + accessory duct opens in the CHD.
  - type I + accessory duct opens in the RHD.
− Type VI: individually, segments II and III drain into the CHD or RHD.
− Type VII: complex variation or unclassified.

### 2.8. Intraoperative Cholangiography (IOC) 

IOC was performed at the gastroenterology center. A 10–20 mL contrast agent was injected through a 5fr drain into the cystic duct. Interpretations were observed and studied using fluoroscopy guidance before and after the procedure. 

### 2.9. Statistical Analysis 

The accuracy of 3D MRCP for the determination of ramification patterns followed by the bile ducts at the hepatic hilum was associated with the gold standard IOC for determining the specificity, sensitivity, negative predictive value (NPV), accuracy, and positive predictive value (PPV) of MRCP as the sole preoperative method for assessing intrahepatic biliary anatomy of living liver donors. 

## 3. Results 

Our study included 35 subjects (29 males and 6 females; age range, 20–45 years; and mean age, 32.5 ± 8.6 years) 

### 3.1. Intraoperative Cholangiography (IOC) Findings 

According to the IOC, a typical intrahepatic biliary pattern (type I) was seen in 23 subjects (62.8%), the type II variant was observed in 4 subjects (11.4%), the type III variant was observed in 4 subjects (11.4%), the type IV was observed in 3 subjects (8.6%), and 1 subject showed unclassified types (2.9%) (Table 2). 

### 3.2. 2D and 3D MRCP Findings 

**Figure 1:**
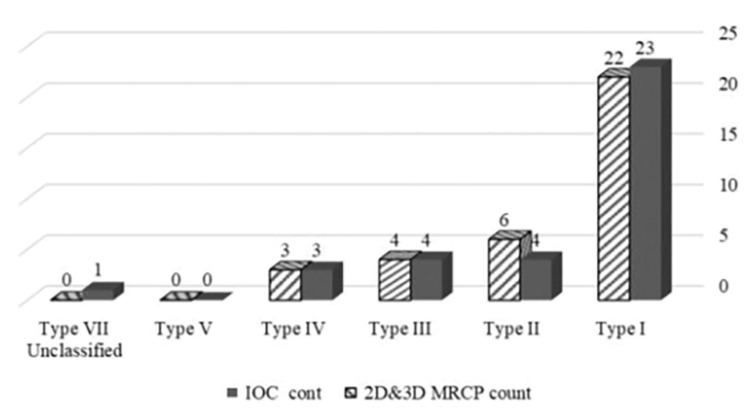
Pars presentation of diagnostic accuracy of MRCP and IOC.

The 2D and 3D MRCP successfully depicted the precise intrahepatic anatomy in 33 out of 35 subjects, but failed to recognize the detailed anatomy in 2 subjects. In the first subject, the RPSD opened in the very short right hepatic duct by IOC, but in the MRCP, it appeared as a trifurcation pattern. In the second subject, the RPSD opened in the LHD by IOC, but in MRCP, it appeared as a trifurcation, probably due to the acute angle between the RPSD and RASD (Table 2 and Figure 1). 

### 3.3. Correlation of MRCP Findings with IOC Findings

MRCP findings in all types, when correlated with those of ICO, showed a strong positive relationship between MRCP and ICO (r = 0.99, 95% CI = 0.87–0.100, *p* = 0.001). 

### 3.4. 3D and 2D MRCP Diagnostic Accuracy 

Using IOC findings as a reference standard, the overall accuracy and sensitivity related to MRCP for the detection of the detailed anatomy of the biliary system were 100% and 94.3%, respectively. 

**Figure 2:**
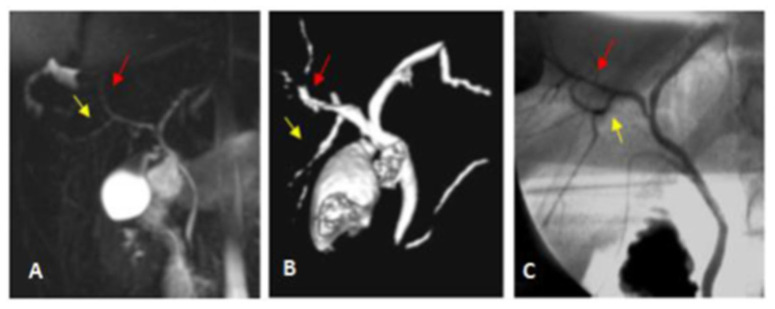
Classic type I biliary anatomy. (**A**) Coronal oblique thin slap heavy T2 FAT SAT FSE image shows RPSD (yellow arrow) joining the RASD (red arrow) to form the RHD. (**B**) Superior view of the post-processed 3D VR coronal oblique image shows RPSD draining into the RHD. (**C**) IOC AP view image confirms the MRCP findings. It shows a small IHB duct of segment VI joining the small IHB duct of segment VII to form the RPSD (yellow arrow), which joins the RASD (red arrow) to form the RHD at a distance > 1 cm from the confluence of both hepatic ducts.

**Figure 3:**
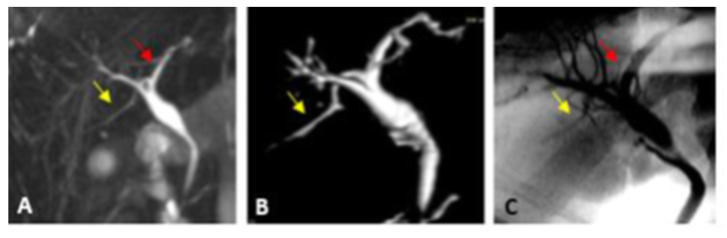
Type III-a variant intrahepatic biliary anatomy. (**A**) Coronal oblique thin slap heavy T2 FAT SAT FSE image shows RPSD (yellow arrow) draining into the LHD (red arrow). (**B**) Antero-posterior view of the post-processed 3D VR coronal image reveals insertion of the RPSD into the LHD. (**C**) IOC AP view image confirms the MRCP findings. They show insertion of the RPSD (yellow arrow) into the distal part of the left hepatic duct (red arrow).

**Figure 4:**
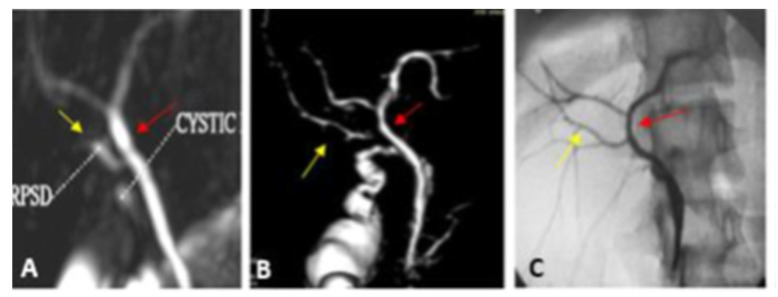
Type III-b intrahepatic biliary variant. (**A**) 2D coronal oblique thin slap heavy T2 FAT SAT FSE image. (**B**) Anterior view of post-processed 3D VR coronal image shows the insertion of the RPSD (yellow arrow) into the middle part of the CHD (red arrow) above the cystic duct. (**C**) IOC AP view image confirms the MRCP findings and shows the insertion of the RPSD (yellow arrow) into the middle part of the common hepatic duct.

**Figure 5:**
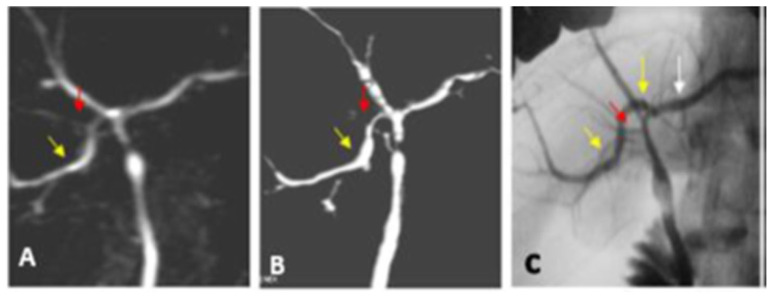
Type VII unclassified intrahepatic biliary variant. (**A**) Oblique view of post-processed coronal 2D (MIP) image. (**B**) Anterior view of post-processed 3D VR coronal image shows the RPSD (yellow arrow) draining into the confluence of the RHD and LHD (reported as trifurcation). Small right accessory duct (red arrow) draining into the proximal part of the common hepatic duct. (**C**) The IOC AP view image shows the insertion of the RPSD (yellow arrows) into the distal end of the left hepatic duct (white arrow). The small right accessory bile duct (red arrow) is seen draining into the proximal common hepatic duct.

Representative cases of our study are illustrated in Figure 2, Figure 3, Figure 4 and Figure 5.

## 4. Discussion 

The biliary tree has a high frequency of variations; as a result, misidentification of the biliary anatomy can result in several complications that may influence the patient’s initial prognosis. Biliary anatomy analysis is critical for the surgical results of liver procedures, especially in cases of liver transplantation. Failure to recognize any minor intrahepatic branches that cross the dissection line can ultimately result in unembellished postoperative biliary leakage and complications. Biliary complications following liver transplantation have been stated in 10–25% of cases overall, with approximately 10% of these cases proving fatal [17,18]. Over the last two decades, MRCP has evolved considerably, aided by improvements in acquisition speed and spatial resolution. It now has a recognized role in examining many disorders related to the biliary nature, helping as a non-invasive substitute for ERCP. MRCP uses heavily T2-weighted pulse sequences in which slow-moving or static fluids inside the pancreatic duct and the biliary tree appear as high signal strength on MRCP. Simultaneously, the adjacent tissue has a reduced signal intensity. Heavily T2-weighted images were initially obtained via modified fast spin-echo (FSE) sequences and gradient-echo (GRE) balanced steady-state free precession techniques [18,19,20,21].

This study adopted the Choi et al. classification [3] of biliary branching patterns. Type I (classic) showed horizontal RPSD joining the vertical RASD to form RHD, joining the confluence with a stem more than 1 cm in length; type II: trifurcation pattern; type III: RPSD inserted into LHD (a), into CHD (b), and into the cystic duct (c); type IV: RHD inserted into the cystic duct; type V: classic type with accessory duct inserted into CHD (a) or RHD (b); type VI: individual draining of segments II and III into RHD; and type VII: unclassified patterns. Wang et al. [22] showed that 56% of the donors had a type I classic branching pattern, 11% had a type II trifurcation pattern, 18% had a type III branching pattern, and 8% had a type III-b branching pattern. In a study conducted by Basaran et al. [23], 67.5% of donors presented with a type I classic branching pattern, 5% with a type II trifurcation pattern, 20% with type III-a, and 2.5% with type III-b. Uysal et al. [24] studied 1011 potential donors using MRCP. The type I classic branching pattern was observed in 803 subjects (79.4%), while the remaining 208 subjects (20.6%) presented different anatomical variations. Düşünceli et al. [25] reviewed MRCP examinations of 475 patients with suspected pancreaticobiliary disorders and found 115 individuals (24.2%) with various anatomic abnormalities. Our study yielded similar results. According to the IOC, 23 out of 35 subjects (65.7%) had a type I classic branching pattern, 4 (11.4%) had a type II trifurcation branching pattern, 2 (5.7%) had a type III-a pattern, 2 (5.7%) had a type III-b pattern, 3 (8.6%) had a type IV pattern, and 1 (2.9%) had a type VII unclassified pattern. MRCP revealed type I in 22 subjects (62.9%), type II variant in 6 subjects (17.1%), type III variant in 4 subjects (11.4%), and type IV variant in 3 subjects (8.6%). MRCP could not correctly diagnose type VII, so it was interpreted as type II. Additionally, MRCP falsely diagnosed one of the standard classical types as a pattern. While the IOC precisely delineated the intrahepatic biliary radicles in all subjects, the 3D MRCP accurately delineated 33 subjects, with a diagnostic accuracy of 97.1%. 

Sodickson et al. [14] discussed the main advantages of 3D isotropic MRCP over standard 2D MRCP; they concluded that the former has relatively thin sections without any gaps at intersections. The data set of 3D allows the postprocessing software to generate any projection of desire and clarify subtle anatomical features that might not be noticeable in traditional 2D standard images. Many studies have shown that 3D MRCP outperforms 2D thick-slab imaging in depicting the anatomy of the nondilated bile duct. 2D MRCP appears to be inadequate for identifying intrahepatic biliary ducts. In the thick- and thin-slab 2D SSFSE imaging, only a minor percentage of the right anterior and posterior branches were observed (12% and 35%, respectively), with no depiction of the lateral and left medial branches. Numerous characteristics limit the depiction of the biliary tree using the standard conventional 2D MRCP technique, particularly in depicting nondilated ducts [26,27,28]. The major drawback of 3D imaging is the acquisition time of 5 minutes, which is longer than the relatively short 2D acquisition time duration. Coronal reconstruction is preferable for visualizing and evaluating the bile ducts [29]. The current study employed 2D and 3D MRCP to map the biliary anatomy. More important anatomical details were provided by the 3D MRCP of the nondilated bile branches with improved spatial resolution and higher accuracy in the results involving the second and third branching patterns than by the 2D MRCP alone. 

The current study confirms the accuracy of 3D MRCP in depicting the biliary anatomy in living liver donors. MRCP revealed classic intrahepatic biliary anatomy in 22 subjects compared to IOC, which revealed 23 subjects with a diagnostic sensitivity of 100%, an accuracy of 95.7%, a specificity of 95.7%, an NPV of 100%, and a PPV of 95.7%. Regarding intrahepatic biliary variants, MRCP detected 11 of 12 subjects with a diagnostic accuracy of 91.7%, specificity of 95.8%, 100% sensitivity, 100% NPV, and 91.7% PPV. Overall, MRCP precisely mapped intrahepatic biliary radicles in 33 of 35 subjects, with a diagnostic accuracy of 94.3%, a sensitivity of 100%, and a PPV of 94.3%. Our results are similar to those of many other studies that reported that MRCP could be used to provide a non-invasive preoperative evaluation of the biliary tract anatomy. Ragab et al. [16] examined the 3D MRCP and IOC data of 20 consecutive liver donors. They showed that the biliary anatomy of 18 donors was compatible and that the specificity and PPV of 3D MRCP for determining the normal biliary anatomy were 100%. Limanond et al. [30] used standard MRCP with a T2WI SSFSE sequence in the preoperative mapping of the biliary tracts of 26 living liver donors. Although only a limited number of subjects were included as samples, the overall MRCP diagnostic accuracy was 84.6%, with 89.5% sensitivity for normal subjects and 71.4% for variants. Kim et al. [31] evaluated liver donors anatomically. In this evaluation, when a comparison was made with the definite anatomy of the biliary components on the IOC, the accuracy of MRCP was 90%. MRCP achieved correct delineation of normal anatomy in 15 out of 17 patients and anomalous anatomy in 12 out of 13 patients. In a study by Donmez et al. [32], 3D MRCP was able to accurately determine the correct biliary anatomy in 32 out of 36 cases, with sensitivity, specificity, PPV, and NPV values of 96%, 72.7%, 88.9%, and 88.9%, respectively. Lee et al. [33] reported an accuracy of 81.8% for MRCP in preoperative biliary mapping in 11 liver donors.

MRCP data collection has been significantly improved in terms of spatial and temporal resolution, allowing MRCP to remain the gold standard for evaluating hepatobiliary disease. Furthermore, MRCP continues to play a crucial role in the non-invasive evaluation of several pancreaticobiliary disorders [34,35]. Biliary obstruction is one of the best indications for MRCP. MRCP can definitely see dilated biliary ducts and may be used to measure the extent of obstruction in extrahepatic bile ducts. Furthermore, the great spatial and contrast resolution provides preoperative information on the intra- and extra-biliary distribution of potentially malignant strictures [35,36,37,38]. 

The limitations of the study include its small sample size and the fact that only prospective liver donors were included.

## 5. Conclusions 

Delineation of the precise normal biliary anatomy and anatomical variants is mandatory for donor selection and surgical planning to minimize postoperative biliary complications in the donor as well as the recipient. MRCP (2D and 3D) is a reliable method for preoperative assessment of the normal intrahepatic biliary anatomy and anatomical variants; nevertheless, adding contrast-enhanced MRCP will significantly improve the diagnostic accuracy and approximate it to the golden standard IOC.

## Figures and Tables

**Table 1:** Scanning parameters of MRCP.

Scanning Parameters	2D T2 SSFSE Axial, Coronal	3D Heavy T2 FAT SAT Coronal Oblique	2D Thick SLAP Myelo Coronal
TE ms	120	480	1200
TR ms	1050	1860	3120
FOV	40	32	34
Slice thickness	4	3	4
NEX	1	2	1
Frequency	256	384	320
Flip angle	150	180	180
Scan time (minutes)	1.39/1.11	2.33	30–40 s

MRCP = magnetic resonance cholangiopancreatography; TE = echo time; TR = repetition time; FOV = field of view; NEX = number of excitations; ms = milliseconds.

**Table 2:** The intrahepatic biliary anatomy based on 2D and 3D MRCP and IOC.

	Description	IOC	MRCP	Probable Cause of Misdiagnosis
Type I	The RPSD drains into the proximal part of the RHD (distance more than 1 cm from the hepatic confluence)	23 (65.7)	22 (62.9)	Very short RHD
Type II	Trifurcation	4 (11.4)	6 (17.1)	
Type III	Anomalous RPSD insertion	4 (11.4)	4 (11.4)	
A	Insertion of RP into the LT hepatic duct	2	2	
B	Insertion of RP into the CHD	2	2	
C	Insertion of RP into the cystic duct	-	-	
Type IV	Insertion of RHD in the cystic duct	3 (8.6)	3 (8.6)	
Type V	Type I +Presence of accessory duct	-	-	
A	Insertion of the accessory duct into the CHD	-	-	
B	Insertion of the accessory duct into the RHD	-	-	
Type VI	Individual draining of segments II and III ducts into RHD	-	-	
Type VII Unclassified	Trifurcation + accessory duct	1 (2.9)	-	The acute angle between RPSD and RASD

The data represent the number of subjects with the percentage in parenthesis. MRCP = magnetic resonance cholangiopancreatography; IOC = intraoperative cholangiography; RP = right posterior; RA = right anterior; RHD = right hepatic duct; LHD = left hepatic duct; CHD = common hepatic duct; RPSD = right posterior sectional duct.

## Data Availability

The datasets used and/or analyzed during the current study are available from the corresponding author upon reasonable request.
